# Supplementary material for: Tube–Iris Distance and Corneal Endothelial Cell Damage Following Ahmed Glaucoma Valve Implantation
Source: J Clin Med. 2022 Aug 28;11(17):5057. doi: 10.3390/jcm11175057 (PMC9456630; doi:10.3390/jcm11175057)
Supplement: Supplementary file 1 [file jcm-11-05057-s001.zip › jcm-1832513-supplementary.pdf]

Supplementary Materials

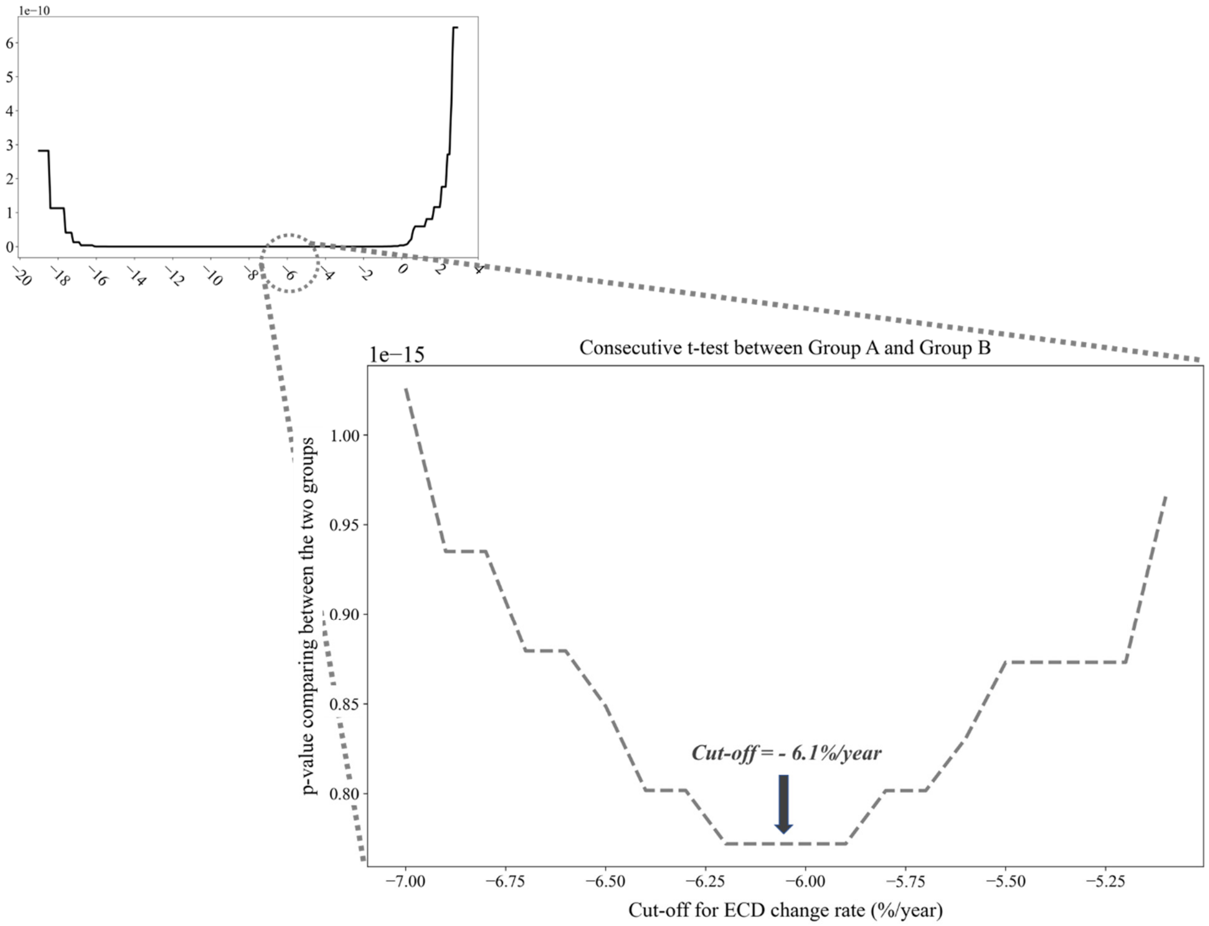

Supplementary Figure S1. Results of consecutive t-tests determining the optimal cut-off value for the endothelial cell density (ECD) change rate. ECD change rates were separated into two groups according to the evaluated cut-off values, and t-tests were conducted to compare the means of the ECD change rates between these groups. Using this methodology, we determined an optimal cut-off value of -6.1%/year, as this value had the smallest associated p-value.

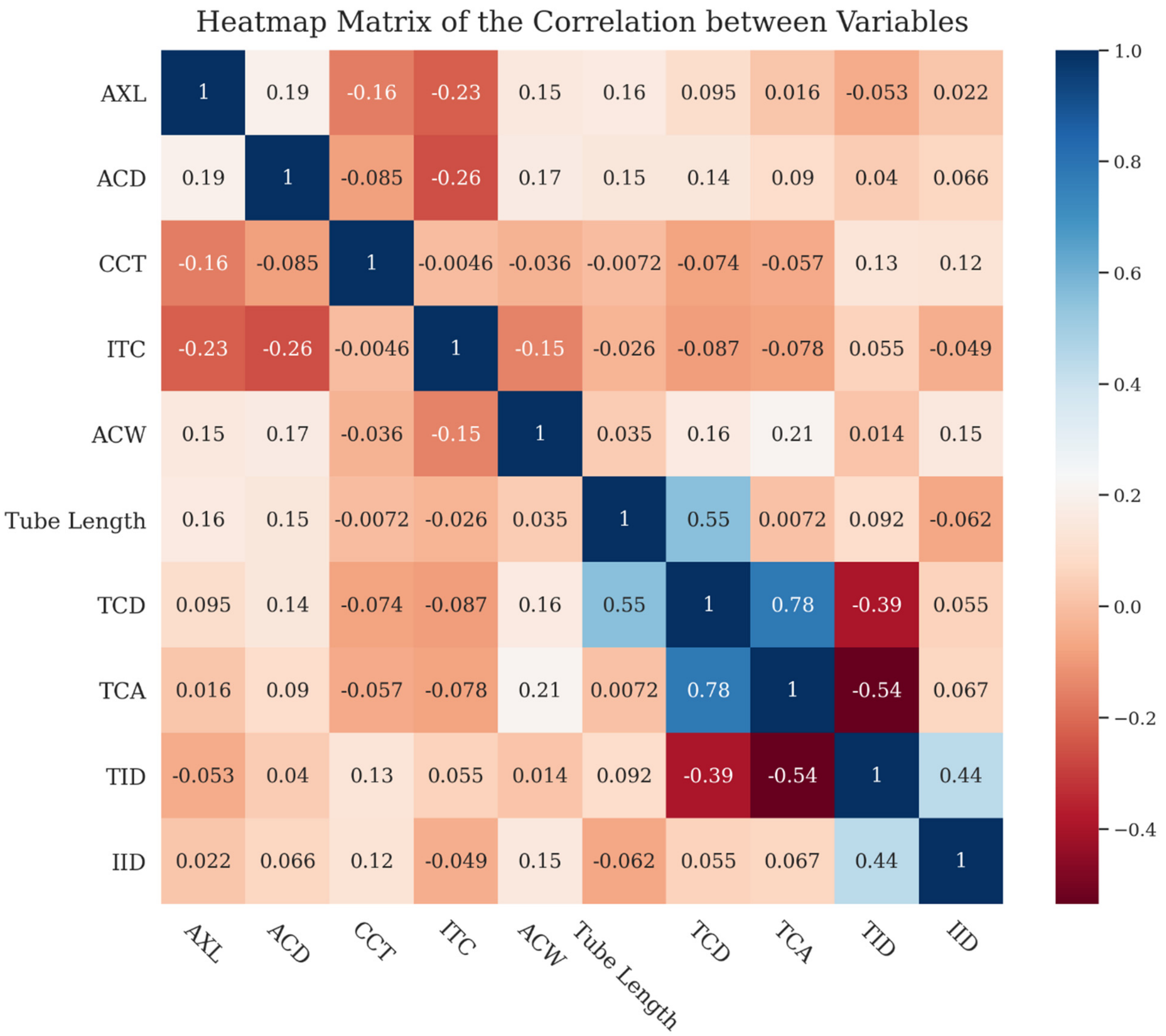

Supplementary Figure S2. Correlation heatmap matrix between a range of independent variables. Pearson's r was calculated between each pair of independent variables to verify collinearities.

Abbreviations: ACD, anterior chamber depth; ACW, anterior chamber width; AXL, axial length; CCT, central corneal thickness; IID, insertion-iris distance; ITC, iris trabecular meshwork contact; TCA, tube-corneal angle; TCD, tube-corneal distance; TID, tube-iris distance

Supplementary Table S1. Comparison of postoperative IOP and uveitis incidence between patients with short TID and long TID.

	**TID < 0.33 mm (n=66)**	**TID ≥ 0.33 mm (n=37)**	**p-value**
**Final IOP (mmHg)**	14.36 ± 4.01	13.83 ± 5.34	0.569*
**Postop Uveitis**	1 (1.52)	0 (0.00)	1.000†
	**TID < 0.371 mm (n=70)**	**TID ≥ 0.371 mm (n=33)**	**p-value**
**Final IOP (mmHg)**	14.33 ± 4.03	13.84 ± 5.45	0.612*
**Postop Uveitis**	1 (1.43)	0 (0.00)	1.000†

*t-test; †Fisher's exact test

IOP, intraocular pressure; TID, tube-iris distance.

Supplementary Table S2. Combinations of variables related to tube-corneal angle were used for additional univariable linear regression analyses.

	Univariable analysis		
	Coefficient	95% CI	p-value
TCD	-0.0040	[-0.0441, 0.0360]	0.842
TCA	0.0011	[-0.0005, 0.0027]	0.173
TCD/TCA	1.7264	[-3.7824, 0.3297]	0.099
TCA/IID	0.0002	[-0.0003, 0.0006]	0.393
IID/TCA	-1.2467	[-2.3017, -0.1918]	0.021*
TCA/AXL	0.0259	[-0.0129, 0.0646]	0.188
AXL/TCA	-0.0284	[-0.0706, 0.0138]	0.185
TCA/ACW	0.0126	[-0.0064, 0.0317]	0.192
ACW/TCA	-0.0572	[-0.1491, 0.0346]	0.219

Only IID/TCA showed significance for predicting ECD change rate (%/year)

CI, confidence interval; TCD tube-corneal distance, TCA tube-corneal angle, IID insertion-iris distance, AXL axial length, ACW anterior chamber width, *p<0.05
